# Hereditary neuropathy with liability to pressure palsies misdiagnosed as Guillain–Barré Syndrome: A case report

**DOI:** 10.1097/MD.0000000000030768

**Published:** 2022-09-23

**Authors:** Jianming Zhu, Xueqing Tong, Yandeng Li, Guangqin Li, Zhendong Pi

**Affiliations:** a Department of Neurology, The First People’s Hospital of Changde City, Changde, Hunan, China; b Department of Nursing, Changde Vocational Technical College, Changde, Hunan, 415000, China.

**Keywords:** electromyography, genetic testing, Guillain–Barré Syndrome, HNPP, PMP22

## Abstract

Hereditary neuropathy with liability to pressure palsies (HNPP) is an autosomal dominantly inherited genetic disease characterized by recurrent numbness and limb weakness. HNPP can be easily missed or misdiagnosed because of electrophysiological heterogeneity and atypical clinical symptoms. To date, diagnosis of HNPP remains a challenge for clinicians.

**Patient concerns:**

Here, we report the case of a 12-year-old woman diagnosed with HNPP, which was initially diagnosed with Guillain–Barré Syndrome (GBS) and treated with intravenous immunoglobulin (IVIG).

**Diagnoses:**

Repeat electrodiagnostic studies and genetic testing confirmed the diagnosis of HNPP.

**Interventions:**

The patient was treated with neurotrophic drugs and health education, including avoiding maintenance of a certain posture for extended periods, which could damage the peripheral nerves.

**Outcomes:**

The patient was discharged 5 days later. The patient was free from recurrence after 6 months of follow-up.

**Lessons:**

This case highlights the complexity of HNPP diagnosis and emphasizes the importance of early identification.

## 1. Introduction

The clinical presentation of hereditary neuropathy with liability to pressure palsies (HNPP), such as limb numbness, fatigue, and/or pain, is variable. And electromyography lacks specificity. There is a need to distinguish HNPP from other diseases, such as Guillain–Barré Syndrome (GBS) or diabetic peripheral neuropathy, and it can be easily missed or misdiagnosed. In turn, this may increase patients’ financial burden and lead to doctor–patient contradictions. Here, we present a case of HNPP misdiagnosed as GBS. We also reviewed the available literature on the pathogenesis, clinical manifestations, and examination and treatment of HNPP. This study aimed to improve the awareness of HNPP and reduce the rate of misdiagnoses and missed diagnoses.

## 2. Case report

The patient was 12 years old, junior school student with no medical history. She was referred to our hospital in November 2021 because of recurrent right upper limb weakness that had persisted for 4 years. Symptoms started to appear at the age of 8 years with the right upper limb having predominantly proximal muscle weakness, which improved after 1 to 2 weeks. The patient’s quality of life was unaffected during the interictal period. She first sought psychiatric help before 6 months, and then a full diagnostic work-up, including serum tests, magnetic resonance imaging (brain, cervical spine), and cerebrospinal fluid cytology, was negative. Electrophysiological examination suggested a demyelinating neuropathy. As a result, she was initially diagnosed with GBS, treated with intravenous immunoglobulin (IVIG), and discharged in a stable condition after 5 days. She presented with recurrence of right upper limb weakness two weeks prior to presentation. Additionally, the patient’s father had a previous diagnosis of GBS.

Neurological examination showed weakness in the right upper extremity (Medical Research Council grades were 4a for proximal and 5 for distal muscles) and functional limitation of the upper limb. Her grip was vigorous. The muscular strength of the remainder of the limb was normal, and the tendon reflex was absent. In addition, signs of meningeal irritation and the Babinski reflex were also negative.

Electrophysiological examinations were repeated. Data from nerve conduction velocities suggested multiple neurological abnormalities. Prolonged distal motor latencies and slow conduction velocities were also observed (Table 1). The F-wave mean latency was prolonged, while the F-wave occurrence rate was reduced (Table 2). Needle electromyography showed a prolonged duration of voluntary motor unit potential (MUP) of the right deltoid muscle and weakened active recruitment response.

**Table 1:** Results of the nerve conduction study.

	Left (right)			
	Lat(ms)	Amp (mV)	Dist (mm)	CV (m/s)
**Motor conduction velocity**				
Ulnar nerve				
Wrist-ADM	2.87(2.31)	10.2(11.2)	–	–
Wrist-Below the elbow	6.50(6.21)	12.0(11.4)	185(180)	51.0(46.2)
Across the elbow	9.58(9.19)	8.2(11.4)	100(110)	32.5↓(36.9↓)
Musculospiral nerve				
Forearm-EDC	1.75(2.18)	5.2(5.6)	–	–
Above the elbow-forearm	4.79(5.33)	4.6(5.3)	265(170)	54.3(54.3)
Median nerve				
Wrist-APB	3.71(3.65)	19.0(14.6)	–	–
Elbow-wrist	8.00(7.81)	18.0(14.0)	215(210)	50.1(50.5)
Axillary nerve				
Erb-Deltoid	4.46(4.13)	16.2(8.9)	–	–
**Sensory conduction velocity**				
Ulnar nerve				
Finger V-wrist	2.15(2.01)	12.5(14.6)	90.0(85.0)	41.9↓(42.3↓)
Musculospiral nerve				
EPL tendon-wrist	1.60(1.96)	19.0(18.8)	100(100)	62.5(51.0)
Median nerve				
Finger I-wrist	1.97(2.17)	23.8(22.4)	85.0(90.0)	43.1↓(41.5↓)
Figer III-wrist	2.41(2.48)	23.8(23.9)	115(120)	47.7↓(48.4↓)

ADM = abductor digiti minimi, APB = abductor pollicis brevis, EDC = extensor digitorum communis, EPL = extensor pollicis longus; Erb: 2-3cm above the clavicle; ↓: below the normal value.

**Table 2:** Results of F-wave study.

	Lfte (right)			
	F%(%)	F-lat(ms)	M-lat(ms)	CV(m/s)
**Ulnar nerve**				
Wrist-ADM	95.0(75.0↓)	32.5↑(32.3↑)	1.0(1.0)	–
**Median nerve**				
Wrist-APB	100(55.0↓)	27.4(28.9)	2.6(3.1)	–

ADM = abductor digiti minimi, APB = abductor pollicis brevis; ↓: below the normal value; ↑: above the normal value.

**Figure 1.**
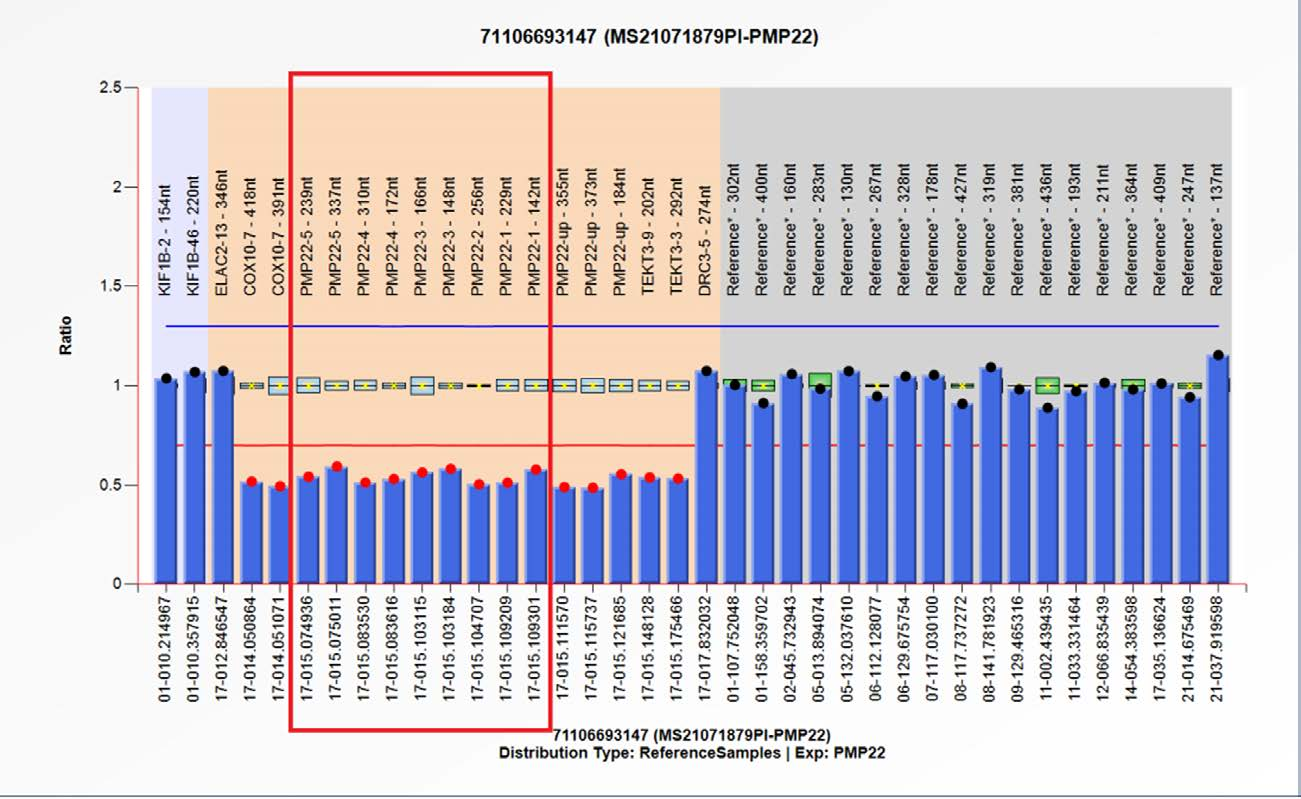
Gene sequencing results showing that this patient carried a heterozygous deletion of the PMP22 gene (deletion of exons 1–5). Note: fluorescence signal intensity less than 0.75 indicates deletion and fluorescence signal intensity greater than 1.25 indicates duplication (0.75-1.25 is the normal range). PMP22 = peripheral myelin protein 22.

In contrast, genetic testing revealed deletion of the peripheral myelin protein 22 (PMP22) gene region on chromosome 17, confirming the diagnosis of HNPP (Fig. 1). Thereafter, we determined the following treatment options: avoiding maintaining a certain posture for extended periods, which would damage the peripheral nerves, and the use of neurotrophic drugs. The patient was free from recurrence after six months of follow-up. Written informed consent was obtained from the patient for publication of the data, and the study was approved by the Research Ethics Board of the First People’s Hospital of Changde City.

## 3. Discussion

HNPP is an autosomal dominantly inherited genetic disease caused by a heterozygous deletion or point mutation of the PMP22 gene in human chromosome 17p12. Deletion or point mutation of the PMP22 gene leads to abnormal expression or function of the PMP22 protein, which is essential for the formation and maintenance of peripheral nerve myelin and protects these peripheral nerves from repeated minor entrapment stimulation. In general, HNPP can occur at all ages, especially at 20 to 30 years old, and there is no significant difference between men and women. In addition, patients with HNPP usually have a positive family history. The prevalence of HNPP has been reported to be at least 16 per 100,000.^[1]^ The age of onset in this case was less than 20 years, which may be related to the differences in the expression of the PMP22 gene in different ethnic populations,^[2]^ which requires further analysis.

The clinical manifestations of HNPP are diverse, and can present as either mononeuropathy or polyneuropathy. Its typical clinical features include recurrent painless muscle weakness and paresthesia.^[3]^ The causes of HNPP include traction, compression, or trauma. The HNPP is self-limiting, with full symptomatic recovery within days, weeks, or months.^[4]^ In addition, very few patients have partial neurological deficits, which makes diagnosis difficult. In this report, we analyzed a case in which the symptoms improved after diagnostic gamma globulin therapy at an outside hospital, which was associated with self-limitation of the disease rather than drug activity. Olivia et al reported that pain occurs in 15% of patients, while Sophie reported a 52% prevalence of neuropathic pain in patients with HNPP.^[5]^ Therefore, pain was not excluded. Among the single nerve lesions in HNPP, the ulnar, radial, median, and common peroneal nerves are the most common, followed by the brachial plexus. Previous data have shown that peroneal or ulnar nerve palsy occurs in approximately 70% of patients, while brachial plexus damage occurs in 10% to 25% of patients.

Because of the diverse clinical features of HNPP, especially in its early stages, there is a high rate of misdiagnosis and missed diagnoses. Electrophysiological examination is an important tool in the diagnosis of HNPP.^[6]^ The intensity of electrophysiological examinations may not be related to the severity of clinical findings. Typical electrophysiological manifestations of HNPP include generalized sensorimotor neuropathy with diffusely abnormal sensory nerve conduction velocity and slowed motor nerve conduction velocity. Our patient presented with the typical HNPP characteristics. Therefore, electrophysiological examination provides better diagnostic efficiency for patients with HNPP with atypical clinical symptoms.

Abnormal PMP22 in HNPP paradoxically leads to focal thickened myelin,^[7]^ which is characterized by sausage-like expansions, referred to as tomacula.^[8]^ With the widespread clinical use of genetic testing and the invasive nature of nerve biopsy, the role of nerve biopsy in the diagnosis of HNPP is limited, and peripheral nerve biopsy is only considered when there is high clinical suspicion of HNPP without positive genetic test results.

Although data on imaging descriptions of the HNPP are limited, they have attracted more research attention.^[9]^ Visser and Beekman first described the ultrasonographic features of HNPP in their case report. They demonstrated nerve enlargement at typical entrapment sites.^[10]^ This conclusion is consistent with the pathological features of HNPP; however, the existence of nerve enlargement outside these sites is controversial.^[11]^ In contrast, Natalie showed that nerve and muscle ultrasound is a useful tool to distinguish between different types of hereditary neuropathy by analyzing nerve and muscle ultrasound parameters in 150 patients with genetically confirmed hereditary neuropathy.^[12]^ Another study reported a case of HNPP confirmed by neurosonography.^[13]^ Therefore, the value of neurosonography for the diagnosis and prognosis of HNPP has been increasing.^[13]^ In addition, the patient in our study showed no evidence of abnormalities on magnetic resonance imaging (MRI) of the head and cervical spine. This finding was consistent with previous literature.^[14]^ However, nerve MRI might not be essential and can be used to further shorten the differential diagnosis.

Currently, no specific therapy is available for HNPP.^[4]^ Treatment is based on neurotrophic drugs as well as symptomatic and supportive therapies. The key to HNPP lies in prevention, mainly to avoid nerve compression caused by exercise or heavy physical work.

## 4. Conclusion

In summary, our analysis showed that the clinical manifestations of HNPP included limb weakness and/or numbness. Patients may have subclinical symptoms, and the scope of electrophysiological examination should be expanded in suspicious cases. The gold standard for the diagnosis of HNPP is identification of PMP22 deletions or point mutations. In addition, HNPP should be diagnosed as early as possible to avoid unnecessary loss due to misdiagnosis.

## Acknowledgments

Thanks to the patient, all the participants and researchers involved in this study.

## Author contributions

**Data curation:** Xueqing Tong.

**Writing – original draft:** Yandeng Li, Guangqin Li.

**Writing – review & editing:** Jianming Zhu, Zhendong Pi.
